# A devised strategy for tracheal extubation for predicted difficult airway in a child with unilateral vocal cord paralysis: a case report

**DOI:** 10.1186/s40981-017-0091-8

**Published:** 2017-05-03

**Authors:** Mariko Nagata, Yasuyo Shimomura, Yoshitaka Hara, Tomoyuki Nakamura, Seiko Hayakawa, Hidefumi Komura, Junpei Shibata, Chizuru Yamashita, Osamu Nishida

**Affiliations:** Department of Anesthesiology and Critical Care Medicine, Fujita Health University School of Medicine, 1-98 Dengakugakubo, Kutsukake-cho, Toyoake, Aichi 470-1192 Japan

**Keywords:** Vocal cord paralysis, Vocal fold paralysis, Difficult tracheal extubation, Airway exchange catheter, Endoscope examination

## Abstract

**Background:**

Extubation is a more challenging medical practice than intubation, and countermeasures against it are similar to those described in the Difficult Intubation Guidelines, but problems cannot be overcome by completely the same methods. We predicted difficult extubation in a pediatric patient with left recurrent laryngeal nerve paralysis and devised an extubation method.

**Case presentation:**

The patient was a 2-year-and-8-month-old boy scheduled for cleft palate repair. Concomitant cardiac anomaly and first and second branchial arch syndrome-associated facial malformations, such as mandibular micrognathia and auricular malformation, were observed. He had a past medical history of difficult intubation and respiratory arrest on a catheter test under intravenous sedation at 4 months old. Left recurrent laryngeal nerve paralysis was discovered on preoperative examination of the cleft palate, based on which difficulty in postoperative extubation was predicted. A catheter for tracheal tube exchange proposed by the extubation guidelines of the Difficult Airway Society (DAS) was placed, endoscopic examination was performed while inducing spontaneous breathing and swallowing reflex by an otolaryngologist, and the tube was removed while movement of the tissue around the glottis was visually evaluated. The patient was managed in an ICU after extubation, and both the systemic and respiratory conditions were favorable.

**Conclusions:**

Extubation and airway management could be safely performed by devising extubation while conforming to the DAS guidelines.

## Background

Extubation is a more challenging medical practice than intubation, and countermeasures against it are similar to those described in the Difficult Intubation Guidelines, but problems cannot be overcome by completely the same methods. Extubation is difficult because it is necessary to consider the airway conditions including excess laryngeal reflex, reduced airway reflex, laryngeal reflex dysfunction, atelectasis, reduced functional residual capacity, and the influence of the surgical procedure, such as airway injury [1]. We encountered a pediatric patient scheduled for palatoplasty in whom recurrent laryngeal nerve paralysis was discovered before surgery, and we devised the extubation strategy.

The patient was a 2 year and 8 months old boy. The height was 74 cm; body weight, 10.8 kg; born at 39 weeks of gestation through normal delivery; birth weight, 2534 g. Complications were cardiac anomaly and first and second branchial arch syndrome (mandibular micrognathia was noted but trismus was absent, left auricular dysplasia syndrome). The patient had past surgical histories at other hospitals of pulmonary artery banding (PAB) at 1 month old, ventricular switch operation, closure of atrial septal defect, and removal of PAB at 4 months old, and pulmonary arterial angioplasty at 1 year and 3 months old. Palatoplasty is normally performed at 1–1.5 years old, but heart surgery preceded in this patient.

After heart surgery, the patient was brought to the pediatric department of our hospital before cleft palate surgery at 1.5 years old. Since he had a past medical history of respiratory arrest and difficult intubation during cardiac catheterization under sedation at 4 months old, endoscopic examination was performed by an otolaryngologist suspecting laryngomalacia. Laryngomalacia was excluded by this examination, but no mobility of the left vocal cord was observed, and the patient was diagnosed with left recurrent laryngeal nerve paralysis. Endoscopic examination was repeated at 2 years and 6 months old, but no improvement of left vocal cord paralysis was observed.

## Case presentation

The following were the risk factors of the patient for predicted difficult airway: (1) Maxillofacial anomaly due to first and second branchial arch syndrome. (2) History of respiratory arrest and difficult intubation during cardiac catheterization under sedation at 4 months old. (3) A diagnosis of left recurrent laryngeal nerve paralysis at 1.5 years by an otolaryngologist.

To deal with expected risks of this patient, a video laryngoscope, laryngeal mask, and tracheotomy kit were prepared in the operative room for difficult airway management (DAM). In addition, endoscopic examination of tissue around the glottis, tracheotomy, and postoperative ICU management were planned for extubation on the assumption of hemorrhage in the oral cavity and stenosis of the pharyngeal cavity accompanying cleft palate closure surgery and aggravation of the respiratory condition due to tracheal intubation-induced vocal cord edema and right (healthy side) recurrent laryngeal nerve paralysis.

On endoscopic examination, observation of vocal cord mobility is necessary to evaluate recurrent laryngeal nerve paralysis. Observation of the moving larynx and glottis in the presence of spontaneous breathing by an otolaryngologist using a bronchoscope after surgery was planned, but there are risks of breath holding and laryngeal spasm in intratracheal operation under sedation. Thus, referring to the extubation guidelines [1] of the Difficult Airway Society (DAS), extubation using a catheter for tracheal tube exchange (airway exchange catheter (AEC)) was planned. DAM was also prepared, and an anesthesiologist was added.

**Fig. 1:**
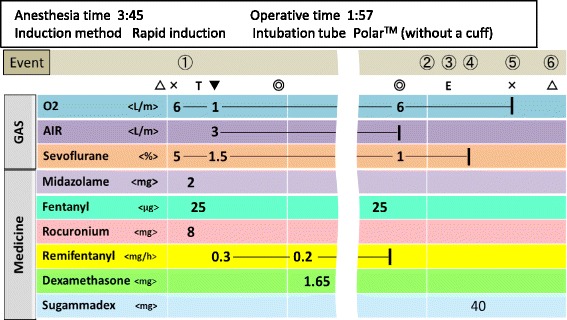
Anesthesia record. Details of events (*1*–*6*). (*1*) Glossoptosis was caused by initiation of sedation, and ventilation became difficult. It was resolved by airway intubation. (*2*) Initiation of spontaneous breathing. (*3*) AEC (Cook Airway Exchanger Catheter™ 8.0 Fr) was inserted and placed, and the tracheal tube was removed. (*4*) An otolaryngologist confirmed the glottis by endoscopy. No problem with the vocal cord or upper airway tissue was noted, and AEC was removed. (*5*) Spontaneous breathing was favorable, but airway obstruction by glossoptosis was noted. Thread to pull the tongue was placed on the tongue tip during surgery, and obstruction was improved by pulling. (*6*) The postoperative course was observed in an ICU. Dexmedetomidine was administered for sedation, but no problem with the respiratory condition occurred, and the patient was transferred to a general ward. (△) Operation room in/out. (×) Anesthesia start/completion. (T) Endotracheal intubation. (▼) Completion of induction. (◎) Surgery start/completion. (E) Endotracheal extubation

**Fig. 2:**
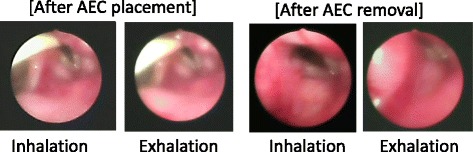
Photographs of bronchoscopic examination

The course of anesthesia is shown in Fig. 1. After securing a route of drip infusion, anesthesia was rapidly induced with midazolame after sedation with oxygen and sevoflurane. Glossoptosis occurred and ventilation became difficult after sedation, but ventilation became easy by oral insertion of oropharyngeal airway, and rocuronium bromide was administered. The larynx could be easily exposed. For the tracheal tube, Polar™ 4.5 mm was orally inserted, the air leakage was confirmed, and a pharyngeal pack with gauze was applied. Anesthesia was maintained with oxygen, air, sevoflurane, and remifentanil. Dexamethasone was administered to prevent laryngeal edema. On completion of surgery, the pharyngeal packing with gauze was removed and the air leakage was less than immediately after the endotracheal intubation. It has been reported that the endotracheal tube air leak test does not predict extubation outcome in pediatric patients [2]. Therefore, we decided to use the AEC as planned. Continuous administration of remifentanil was completed, and fentanyl was administered to prevent postoperative pain and excitement at arousal. The sevoflurane concentration was gradually decreased, and spontaneous breathing started. For AEC, Cook Airway Exchanger Catheter™ 8.0 Fr (O.D. = 2.7 mm, I.D. = 1.6 mm) was prepared. This catheter is hollow, and two side holes are present at the tip, securing sufficient ventilation. AEC was inserted as follows: Firstly, AEC was inserted through the orally inserted tracheal tube, and the tracheal tube was removed. Then, an otolaryngologist endoscopically confirmed the glottis through the nostril. There was no abnormal finding, such as edema, in the upper airway tissue or no problem with mobility of the right (healthy side) vocal cord. AEC was removed while the endoscope was placed. Since mobility of the right vocal cord was also favorable on endoscopic examination after removal of AEC (Fig. 2), the endoscope was removed. Favorable spontaneous breathing continued thereafter, but airway obstruction by glossoptosis occurred. Thread to pull the tongue was placed on the tongue tip during surgery, and obstruction was improved by pulling this thread. The anesthesia time was 3 h and 45 min, and the operative time was 1 h and 57 min. The postoperative course was observed in an ICU while the thread to pull the tongue was left on it. Dexmedetomidine was administered for sedation, but no problem with the respiratory condition occurred, and the patient was transferred to a general ward.

### Discussion

In recurrent laryngeal nerve paralysis, the vocal fold, also known commonly as vocal cord, is immobile during phonation and breathing. Hoarseness and aspiration occur in unilateral paralysis, and dyspnea occurs in bilateral paralysis, for which tracheotomy is necessary.

The causes of pediatric bilateral laryngeal nerve paralysis are mainly neurological, idiopathic, or birth trauma [3]. As for unilateral paralysis, the main cause is iatrogenic. Vocal fold paralysis (VFP) is a known complication of cardiac surgery in children. It has been reported that pediatric VFP occurs after cardiac surgery. In a study with a total of 109 children with VFP, 8 patients had no VFP, 94 patients had left VFP, 6 patients had right VFP, and 1 patient had bilateral VFP. Therefore, the left vocal fold incurs higher impairment compared with the right. This may be explained by the fact that the left recurrent laryngeal nerve presents a longer path, which may be related with ductus arteriosus, making it susceptible to damage during the progression of many diseases and surgical procedures [4].

Tiago et al. have reported a case in a child which, after performing surgery to close the patient’s ductus arteriosus, evolved with breathing difficulties and dysphonia. They suggested that flexible fiber-optic laryngoscopy should be carried out pre- and post-surgery in children for whom heart surgery to correct abnormalities is indicated, thereby allowing for early diagnosis of VFP and the selection of the best management approach [5].

This pediatric patient had a past medical history of difficult intubation and respiratory arrest on catheter test under intravenous sedation at 4 months old. He also received cardiovascular surgery at 1 year and 3 months old, but his parents did not obtain information of respiratory trouble.

Left recurrent laryngeal nerve paralysis was incidentally discovered on preoperative examination before cleft palate surgery at 1.5 years old based on the presence of a past medical history of breathing problem under sedation. The exact onset age of left recurrent laryngeal nerve paralysis is unknown. Aspiration was slightly observed, but his family did not notice recurrent laryngeal nerve paralysis because of the presence of delay in speech and cleft palate.

Since patients do not complain of unilateral recurrent laryngeal nerve paralysis in infancy, it is likely to be overlooked. In such cases, the possibility of recurrent laryngeal nerve paralysis should be taken into consideration from before surgery.

In addition, in cleft palate closure surgery, not only tracheal intubation but also pressing of the tracheal tube by a Dingman mouth gag may damage the recurrent laryngeal nerve. In unilateral recurrent laryngeal nerve paralysis, such as the present case, if the nerve on the healthy side is damaged, the condition becomes bilateral paralysis resulting in dyspnea. Thus, a careful extubation plan is needed.

Various countermeasures against difficult intubation, such as DAM, have been investigated, but no close investigation was performed with regard to extubation. DAS published the extubation guidelines in 2012 [1]. Patients likely to develop complication accompanying extubation are regarded as the high risk group, and the algorithm is comprised of four procedures.

- Step 1: Plan extubation. Assess the airway and general risk factors because the ability to oxygenate is uncertain and reintubation may be difficult, and/or general risk factors may be present. We assessed the risk factors at extubation in the present case, such as maxillofacial anomaly (first and second branchial arch syndrome), hemorrhage in the oral cavity, and stenosis of the pharyngeal cavity accompanying cleft palate closure surgery, as well as aggravation of the respiratory condition due to tracheal intubation-induced vocal cord edema and right (healthy side) recurrent laryngeal nerve paralysis.
- Step 2: Prepare for extubation. DAM was also prepared and an anesthesiologist was added.
- Step 3: Perform extubation. There is a key question, “is it safe to remove the tube?” If the answer is “Yes”, proceed to “Awake extubation” or “Advanced techniques.” Use of laryngeal mask, continuous administration of remifentanil, and extubation with AEC are proposed. If the answer is “No,” “Postponement of extubation” timing and “Tracheotomy” are proposed. We chose “Advanced techniques” and planned bronchoscopic examination during extubation to evaluate recurrent laryngeal nerve paralysis, referring to the DAS extubation guidelines. To evaluate mobility of the vocal cord, intratracheal operation with an endoscope while inducing the spontaneous breathing and the swallowing reflex is necessary, but these may cause breath holding and laryngeal spasm. Thus, in the present case, the AEC was placed following the method proposed by the guidelines, and extubation was performed while an otolaryngologist endoscopically evaluated the moving glottis.
- Step 4: Post-extubation care: recovery and follow-up. After extubation, the patient was managed in the ICU, and the systemic and respiratory conditions were favorable in the postoperative course.

By devising extubation while conforming to the DAS guidelines, extubation and airway management could be safely performed.

## Conclusions

Extubation and airway management could be safely performed by devising extubation while conforming to the DAS guidelines.

## Abbreviations

AEC: Airway exchange catheter
DAM: Difficult airway management
DAS: Difficult Airway Society
PAB: Pulmonary artery banding
VFP: Vocal fold paralysis
